# Processing Method Altered Mouse Intestinal Morphology and Microbial Composition by Affecting Digestion of Meat Proteins

**DOI:** 10.3389/fmicb.2020.00511

**Published:** 2020-04-08

**Authors:** Yunting Xie, Chong Wang, Di Zhao, Guanghong Zhou, Chunbao Li

**Affiliations:** ^1^Key Laboratory of Meat Processing and Quality Control, MOE, Jiangsu Collaborative Innovation Center of Meat Production and Processing, Quality and Safety Control, Key Laboratory of Meat Products Processing, MARA, College of Food Science and Technology, Nanjing Agricultural University, Nanjing, China; ^2^International Joint Laboratory of Animal Health and Food Safety, MOE, Nanjing Agricultural University, Nanjing, China; ^3^National Center for International Research on Animal Gut Nutrition, Nanjing Agricultural University, Nanjing, China

**Keywords:** processed meat proteins, protein digestion *in vivo*, amino acids, gut microbiota, intestinal morphology, SCFAs

## Abstract

Our previous study showed that the intake of meat proteins dynamically affected fecal microbial composition. However, the digestion of processed meat proteins *in vivo* and its relationship with gut microbiota and host remain unclear. In this study, we collected cecal contents and intestinal tissue from the mice fed with casein, soybean protein (SP), and four processed pork proteins for 8 months, and analyzed the amino acid (AA) files, cecum microbial composition and metabolites, and intestinal morphology. Dry-cured pork protein and stewed pork protein (SPP) groups had significantly higher total AA content in gut content than the other groups, but the content of the SPP group was relatively lower in the serum. The microbial composition of the processed meat protein groups differed from the casein or SP group, which is consistent with changes in AA composition. Emulsion sausage protein and SP diets upregulated the microbial AA metabolism, energy metabolism, signaling molecules and interaction, translation, and digestive system function but downregulated the microbial membrane transport, signal transduction and cell motility function compared to the casein diet. The SPP diets increased concentrations of acetate, propionate, butyrate, and isovalerate by specific gut microbes, but it decreased the relative abundance of *Akkermansia.* Moreover, the mice fed SP diet had relatively lower crypt depth, higher villus height and V/C ratio in duodenum, with the longer small intestines and the heavier cecum than other diets. These results suggested that processing methods altered bioavailability of meat proteins, which affected the intestinal morphology and the cecum microbial composition and function.

## Introduction

Meat is an important protein source in diets. However, the proteins in meat are prone to chemical changes during processing (Berardo et al., 2015; He J. et al., 2018), which may affect the digestion and absorption in the host. The gastrointestinal tract is the first place for interplay between dietary nutrients and the host. Previous studies have shown that short-term intake of different dietary proteins caused different gut morphology (Ogura et al., 2008). Gut microbiota has been recognized as an important medium linking food to the host (Derrien and Veiga, 2017). It could be altered by different factors, such as diet, ethnicity, or geography (He Y. et al., 2018). Among these factors, diet has the primary effects on the composition of gut microbiota (David et al., 2014). Some studies have indicated that high-protein diets might alter the gut microenvironment and further affect the gut microbiota (Mu et al., 2016). Thermally processed food significantly affected gut microbiota diversity in vertebrates (Zhang and Li, 2018). Gut microbiota has the capacity of communicating with the host in different ways, so it may affect the host metabolic, nutritional, physiological, behavioral, and immunological processes (Sonnenburg and Backhed, 2016; Blacher et al., 2017). Short-chain fatty acids (SCFAs) are one of the important products of intestinal microbial metabolism, which can enhance the intestinal barrier function and maintain the health of the immune system (Gonçalves et al., 2018). Intestinal peristalsis time determines the use of substances by intestinal microbes (Cryan et al., 2018), and ultimately may affect the contents of SCFAs. An impaired gut microbiota may induce obesity, anorexia nervosa, type 2 diabetes, atherosclerosis, irritable bowel syndrome, and other disorders (Zhang et al., 2010; Koh et al., 2018). Much attention has been paid to the effects of short-term intake of dietary protein on the gut microbiota composition. Our previous study showed that the long-term intake of meat proteins dynamically affected fecal microbial composition (Xie et al., 2019). However, the digestion of processed meat proteins *in vivo* and its relationship with gut microbiota and host remain unclear. Therefore, the objective of this study was to explore the digestion of processed meat protein *in vivo* and its relationship with gut microbiota and intestinal morphology.

## Materials and Methods

### Animals and Diets

Male C57BL/6J mice (4 weeks old) were raised in a specific pathogen-free and controlled environment (60 ± 10% of humidity, 12 h light cycle, 20.0 ± 0.5°C) in an animal center (SYXK < Jiangsu > 2011-0037). Mice were given *ad libitum* access to diet and water. After 2 weeks of adaptation, the animals were divided into six groups (10 mice each group, 2 mice per cage) and fed for 8 months with one of six protein diets, that is, casein (C), soy protein (SP), emulsion-type sausage protein (ESP), dry-cured pork protein (DPP), stewed pork protein (SPP), and steam-cooked pork protein (CPP). Casein and isolated soy protein were obtained from Shanghai Ruian Bio Technologies Co., Ltd. (Shanghai, China) and Linyi Shan Song Biological Products Co., Ltd. (Shandong, China), respectively. Isoflavones were removed from soy protein by 80% methanol. Pork products were made with pork *Longissimus dorsi* muscles from the same carcasses and the specific preparation steps were as described previously (Li et al., 2017) with minor modifications. In brief, cooked pork was prepared according to the following steps: pork loin was cooked in a steam-cooking chamber at 72°C until the core temperature of meat reached 70°C. Emulsion-type sausage was prepared as follows. Pork loin and back fat were mixed at a ratio of 4 to 1, and salt (1.8%) and tripolyphosphate (0.4%) were added. Meat and fat were chopped using a high-speed chopper. Salt and tripolyphosphate were mixed, and the batter was stuffed into 48-mm-diameter plastic casings. The sausage was steam-cooked at 72°C until the core temperature of sausage reached 70°C. Stewed pork was prepared according to the following formulations: pork loin was vertically cut into strips (5 cm width). The strips were blanched, placed in boiling water for about 5 min, chilled and cut into 5 cm × 5 cm × 5 cm cubes. Then, the cubes were pan-fried (180°C) for 5 min with soybean oil (10 g/kg of meat) on an induction surface. They were turned twice at intervals of 60 s and then cooked in boiling water (water/meat: 1/4) for 5 min. Finally, the cubes were stewed at 100°C for 150 min. Dry-cured pork was prepared as follows: pork loin was cured with 5% salt and sun-dried for 1 month. Then, the cured pork was steam-cooked at 72°C until the core temperature of meat reached 70°C. Then, meat proteins were isolated from the above processed pork products by removing fat and water in methylene chloride/methanol solvent (2:1, v: v). The granule and purified type diets were prepared by Trophic Animal Feed High-tech Co., Ltd., China according to the AIN-93G diet formulation (Reeves et al., 1993). The amino acid (AA) profile in protein powders and the ingredient composition and nutritional content of diets were determined following a previous study (Xie et al., 2019), including 20% proteins, 39.75% cornstarch, 13.2% dextrinized cornstarch, 10% sucrose, 7% soybean oil, 5% fiber, 3.5% mineral mix, 1% vitamin mix, 0.3% L-cystine, 0.25% choline bitartrate, and 0.0014% tertbutyl hydroquinone. Body weight and feed intake of mice were routinely recorded during the intervention period and these data can be found following previous study (Xie et al., 2019). All experiments were performed in compliance with the relevant guidelines and regulations of the Ethical Committee of Experimental Animal Center of Nanjing Agricultural University.

### Sample Collection

Mice were euthanized by cervical dislocation after 8 months feeding. The blood samples were collected and centrifuged at 3,000 × *g* for 30 min to pellet the blood cells. Serum samples were collected and stored at −80°C for further analysis. The duodenum, jejunum, cecum, and colon tissues were taken and subjected to fixation in 4% (wt/vol) paraformaldehyde for immunohistochemical analysis. Their luminal contents were immediately frozen in liquid nitrogen and stored at −80°C for subsequent analysis. Moreover, the length of small intestine and colon, and the weight of cecum and its contents were recorded.

### Chemical Analyses

The free AA concentrations were determined by HPLC using an Agilent 1220 Infinity LC system with a fluorescence detector (Agilent Technologies Inc., CA, United States) as previously described (Dai et al., 2014). Briefly, intestinal contents (25 mg) or serum (100 μl) and 1.5 M HClO_4_ (100 μl) were added into a 1.5-ml centrifuge tube and mixed for 1 min. Fifty microliters of 2 M K_2_CO_3_ were added to release CO_2_. Then, samples were centrifuged at 15,000 × *g* at 4°C for 10 min. The supernatant (100 μl) was mixed with 1.2% benzoic acid and saturated K_2_B_4_O_7_ (1.4 ml), and the water phase was passed through a 0.22-μm filter membrane. The filtrate was subjected to react with OPA (o-phthaldialdehyde) solution for 1 min. The resulting samples were loaded onto an HPLC C18 column and eluted by mobile phases of HPLC-grade water (A) and acetonitrile (B). The flow rate was 1.0 ml/min and the column temperature was 30°C. The wavelengths of detector were Ex 340 nm and Em 455 nm, respectively. The SCFAs were observed by gas chromatograph (GC-2010 Plus, Shimadzu, Japan) as previously described (Wang et al., 2011). Briefly, cecal contents (50 mg) were suspended in 250 μl of ddH_2_O and were centrifuged at 12,000 × *g* for 5 min in a micro-centrifuge (Microfuge 22R, Beckman Coulter, CA, United States). The SCFA analysis was completed on the supernatants (200 μl). The internal standard was crotonic acid. The flame ionization detector and a capillary column (Agilent Technologies, HP-INNOWax, 30 m × 0.25 mm × 0.25 μm, CA) were applied. The parameters were set as follows: injector/detector temperature, 180°C/180°C; column temperature, 130°C; and gas flow rate, 30 ml/min.

### Histological Observations

The tissue samples were fixed in 4% paraformaldehyde buffer for 24 h and then were embedded in paraffin. The paraffin-embedded tissues were cut into 5-μm-thick sections by a microtome. The xylene and ethanol (100, 100, 90, 80, and 70%) were used to deparaffinize and rehydrate. Then, the sections were stained in hematoxylin and eosin (H&E) and images were captured by a light microscope (BX51, Olympus, Tokyo, Japan). In each group, 10 mice were used and three sections with 30 HPFs were observed per mouse (i.e., 10 HPFs per section). The morphological parameters were measured using the CellSens Entry software (Olympus, Tokyo, Japan). The villus height was measured from the baseline to the tip of the highest one and the crypt depth was measured from the baseline to the bottom of the deepest one (Gallier et al., 2013).

### 16S rRNA Gene Sequencing

#### DNA Extraction and Gene Sequencing

Total microbial DNA was extracted from the cecal contents using a QIAamp DNA Stool Mini Kit (Qiagen, Germany) according to the manufacturer’s instructions. The DNA was quantified by a spectrophotometer (Nanodrop2000, Thermo, United States). The V4 region of the 16S rRNA genes was amplified by PCR (Wang et al., 2007). PCR was performed in triplicate using the eight-basic sequence unique to each sample (515F 5′-barcode-GTGCCAGCMGCCGCGG-3′ and 806R 5′-GGACTACHVGGGTWTC TAAT-3′). The parameters for the PCR reaction were set to an initial denaturation step at 98°C for 30 s, which was followed by 30 cycles of denaturation at 98°C for 5 s, annealing at 53°C for 20 s, and elongation at 72°C for 20 s, with a final elongation step at 72°C for 5 min. The amplicons were extracted from 2% agarose gel and purified using the AxyPrep DNA gel extraction kit (Axygen Biosciences, CA, United States) according to the manufacturer’s instructions. The amplicons were quantified using QuantiFluorTM-ST (Promega, United States). Library construction and sequencing purified PCR products were quantified by Qubit R 3.0 (Life Technologies). The Illumina Pair-End library was constructed by pooled DNA product following Illumina’s genomic DNA library preparation procedure. Then, the Illumina MiSeq platform was applied to pair-end (2 × 250) sequence amplicon library according to the standard protocols.

Raw fastq files were demultiplexed and quality-filtered using QIIME (version 1.17) with the following criteria. The 250-bp reads were truncated at any site receiving an average quality score <20 over a 10-bp sliding window. The truncated reads were discarded if they were shorter than 50 bp. Sequences were matched and those overlapping longer than 10 bp were assembled (Caporaso et al., 2010). After screening, filtering, and pre-clustering processes, gaps in each sequence were removed in all samples to reduce noise, operational taxonomic units (OTUs) were clustered with ≥ 97% similarity using UPARSE (version 7.1, http://drive5.com/uparse/), and chimeric sequences were identified using UCHIME. The phylogenetic affiliation of each 16S rRNA gene sequence was analyzed by RDP Classifier (http://rdp.cme.msu.edu/) against the SILVA (SSU123) 16S rRNA data with a confidence threshold of 70% (Amato et al., 2013).

#### Bioinformatics Analysis

According to the results of the OTU clustering analysis, the relative abundance of each OTU at different taxonomic levels and calculated diversity indices (Chao, ACE, Shannon index, Simpson index, Good’s coverage) were defined (Schloss et al., 2009). The Bray–Curtis similarity clustering analysis was applied to offer an overview of microbial composition in the cecum (Lozupone et al., 2011). The distance-based analysis of molecular variance (AMOVA) was conducted to further assess the significances between different group samples. To distinguish biological conditions among six diet groups, the linear discriminant analysis effect size (LEfSe) was performed to robustly identify prevalent microbial taxa in each group (Segata et al., 2011). The specific parameters were set as follows: alpha value for the factorial Kruskal–Wallis test among classes and the pairwise Wilcoxon test among subclasses was less than 0.05, the threshold on the logarithmic LDA score above 3.0 that is statistically different between biological classes. The Spearman’s correlation coefficients were assessed to determine the relationships between microbiota and SCFAs. Correlation was considered significant when the absolute value of Spearman’s rank correlation coefficient was greater than 0.6 and *P*-value was smaller than 0.05.

#### Functional Prediction of the Microbial Genes

The Phylogenetic Investigation of Communities by Reconstruction of Unobserved States (PICRUSt) program was applied to predict the functional alteration of gut microbiota (Langille et al., 2013). The OTU data obtained were applied to generate BIOM files formatted as input for PICRUSt v1.1.09 with the make.biom script usable in the Mothur. OTU abundances were mapped to Greengenes OTU IDs as input to speculate about the alteration of microbiota functions.

### Statistical Analysis

Effects of diets were evaluated by one-way ANOVA with the SAS software (SAS Institute Inc., Cary, NC, United States). Means were compared and the significance level was set at 0.05. Figures were made using the GraphPad Prism (version 5.0.3, San Diego, CA, United States).

## Results

### Dietary Proteins Affected AA Profiles in Serum and Gut Luminal Contents

**FIGURE 1:**
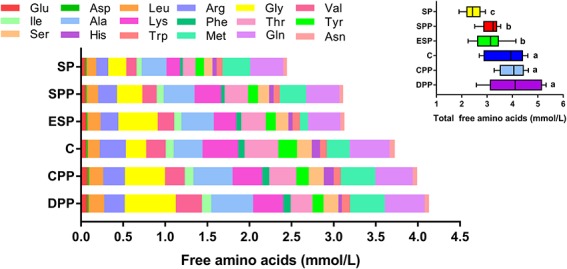
Composition of amino acids in serum. The data were analyzed by one-way ANOVA, and means were compared by Tukey’s *t*-test. The “a, b, c” letters indicate significant differences (*P* < 0.05). C, casein; CPP, cooked pork protein; DPP, dry-cured pork protein; ESP, emulsion-type sausage protein; SP, soy protein; SPP, stewed pork protein.

Mice fed a SP diet had the lowest level of serum total AAs, but the values were much higher for mice fed processed meat protein diets or a casein diet (Figure 1 and Supplementary Table S1). Notably, serum total AAs were higher in mice fed a CPP or DPP diet than in mice fed an ESP or SPP diet. This difference was mainly derived from the quantities of Gly, Val, and Met. All detectable AAs, except Met, His, and Gln, were lower in the SP group than those of casein and processed meat protein diet groups.

**FIGURE 2:**
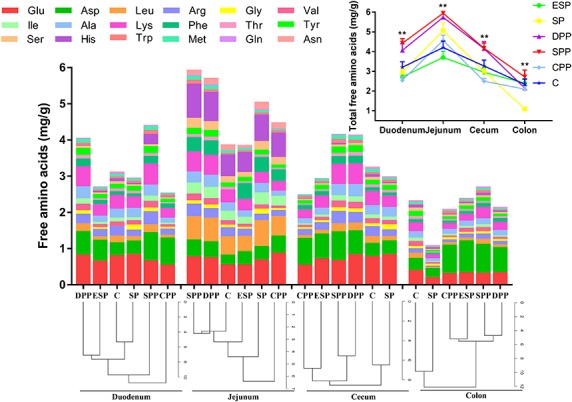
Changes in amino acids in the intestinal contents. The data were analyzed by one-way ANOVA, and means were compared by Tukey’s *t*-test. The asterisks (*) indicate significant differences among diet groups, **P < 0.01. The “a, b, c” letters indicate significant differences (*P* < 0.05). C, casein; CPP, cooked pork protein; DPP, dry-cured pork protein; ESP, emulsion-type sausage protein; SP, soy protein; SPP, stewed pork protein.

In gut luminal contents, protein diets also exhibited a great impact on the total AA contents (Figure 2 and Supplementary Tables S2–S5). The DPP and SPP groups had much higher total AAs in duodenum, jejunum, and cecum (*P* < 0.05), but the other four diet groups did not differ (*P* > 0.05). The values were the lowest for the SP group in colon (*P* < 0.05). No significant difference in the composition of AAs existed between casein and SP groups, or between DPP and ESP groups, or between SPP and CPP groups. However, the processed meat protein groups were significantly different from casein or SP group (Figure 2).

### Dietary Proteins Altered the Development and Morphology of Gut Tissue

**FIGURE 3:**
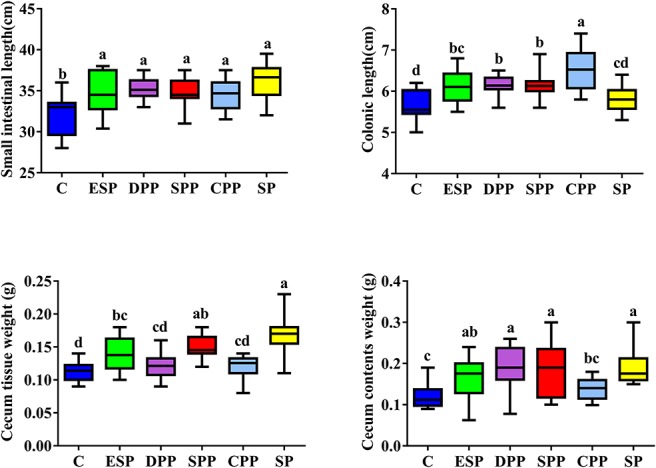
Dietary proteins altered intestinal lengths and cecal sizes. The data were analyzed by one-way ANOVA, and means were compared by Tukey’s *t*-test. The “a, b, c, d” letters indicate significant differences (*P* < 0.05). C, casein; CPP, cooked pork protein; DPP, dry-cured pork protein; ESP, emulsion-type sausage protein; SP, soy protein; SPP, stewed pork protein.

**FIGURE 4:**
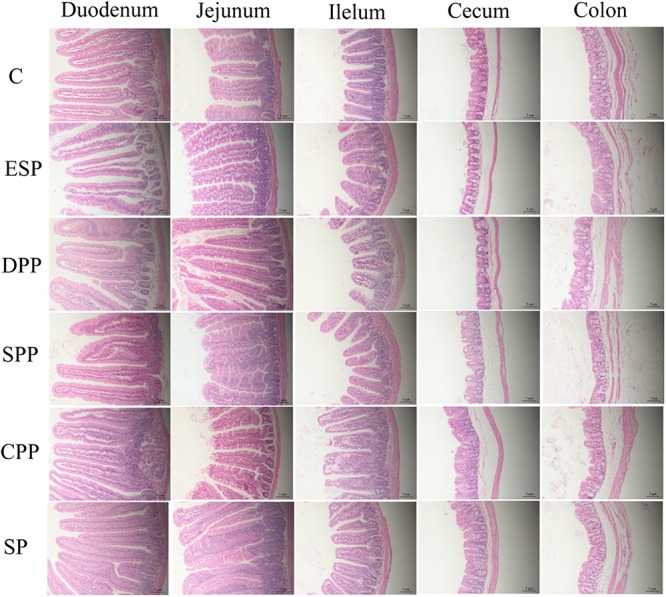
Dietary proteins altered the microstructure of the intestine. The villus height and crypt depth were measured by H&E staining. C, casein; CPP, cooked pork protein; DPP, dry-cured pork protein; ESP, emulsion-type sausage protein; SP, soy protein; SPP, stewed pork protein.

Mice fed casein exhibited the smallest cecum tissue weight and upper intestine length, while the highest values were observed for mice fed a SP. The CPP group had smaller cecum tissue weight but greater colonic length than the other meat protein diet groups (Figure 3). In addition, H&E staining showed significant differences in the tissue microstructure among diet groups (Figure 4 and Table 1). In the duodenum, the SP group had the lowest crypt depth but the highest villus height and V/C ratio (*P* < 0.05). The average value of crypt depth was the greatest for the ESP group. The values of villus height and V/C ratio were the smallest for the casein group. In the jejunum, the ESP group had greater crypt depth, villus height and V/C ratio than the casein group. No significant difference was observed in villus height, crypt depth and V/C ratio in the ileum, or crypt depth in colon. However, in the cecum, the crypt depth was higher in the SP and CPP groups than in other diet groups. These data demonstrate that protein diets significantly influence the development and morphological structure of gut tissue in mice.

**TABLE 1:** Histological parameters of the intestinal tissues.

	**C**	**ESP**	**DPP**	**SPP**	**CPP**	**SP**
**Crypt depth (μm)**						
Duodenum	97.179.84^ab^	103.787.95^a^	91.996.96^b^	95.4614.75^ab^	97.2010.41^ab^	82.586.42^c^
Jejunum	74.1211.28^b^	88.4414.06^a^	91.457.05^a^	75.147.47^b^	90.276.84^a^	84.8912.41^a^
Ileum	69.928.91	72.898.48	65.907.21	62.634.90	66.557.85	66.517.56
Cecum	65.1811.47^b^	62.299.66^b^	65.438.38^b^	66.0215.59^b^	76.4412.06^a^	82.0410.83^a^
Colon	55.2813.74	55.798.83	66.7718.42	62.7213.67	60.5810.12	65.7224.28
**Villus height (μm)**						
Duodenum	345.8059.76^c^	377.2055.44^bc^	400.3140.84^bc^	350.3087.22^c^	406.9477.30^b^	497.5938.64^a^
Jejunum	325.0139.13^c^	434.0085.65^a^	398.6168.66^ab^	392.6976.02^ab^	366.6195.55^bc^	398.6948.81^ab^
Ileum	184.9956.19	211.7421.25	177.3329.24	172.3320.81	183.0746.49	179.0439.32
**V/C ratio**						
Duodenum	3.600.81^c^	3.660.63^c^	4.360.42^b^	3.710.95^bc^	4.240.92^bc^	6.070.80^a^
Jejunum	4.440.60^ab^	5.041.20^ab^	4.390.84^b^	5.261.08^a^	4.061.05^b^	4.740.60^ab^
Ileum	2.620.65	2.930.33	2.710.45	2.760.29	2.730.53	2.680.42

*Values are shown as mean ± SD, and means were compared by Tukey’s *t*-test. The “a, b, c” letters indicate significant differences (*P* < 0.05). C, casein; CPP, cooked pork protein; DPP, dry-cured pork protein; ESP, emulsion-type sausage protein; SP, soy protein; SPP, stewed pork protein.*

### Dietary Proteins Affected the Composition and Function of Cecal Microbiota

#### Richness and Diversity of Microbiota

**FIGURE 5:**
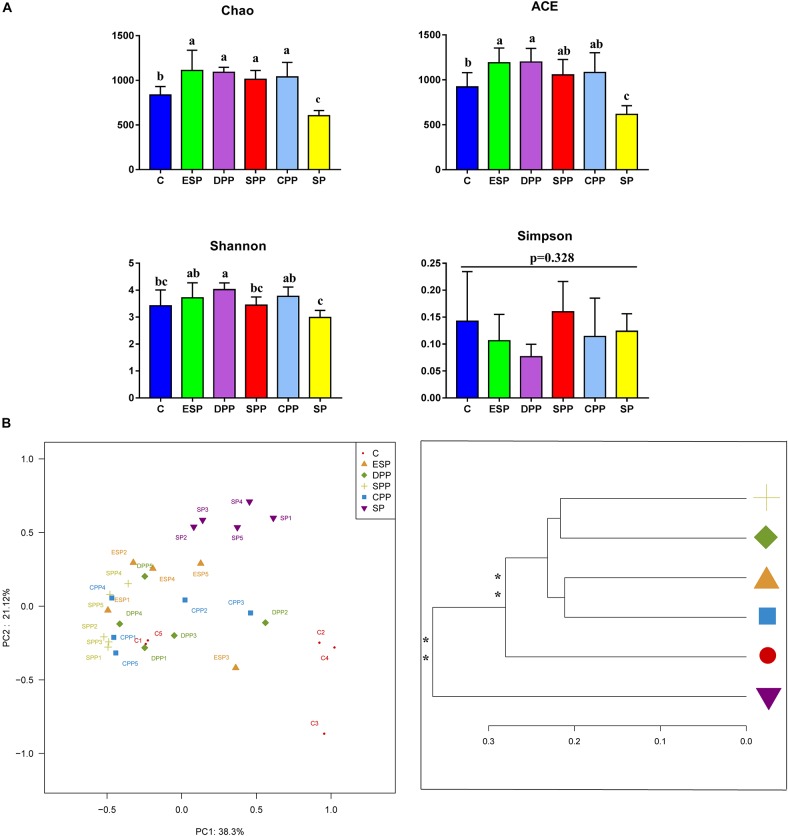
Richness and diversity of cecum microbiota. **(A)** Alpha diversity. **(B)** Principal coordinate analysis and clustering analysis. The data were analyzed by one-way ANOVA, and means were compared by Tukey’s *t*-test. The “a, b, c” letters indicate significances (*P* < 0.05). The MANOVA significance was also indicated: *P < 0.05; **P < 0.01. C, casein; CPP, cooked pork protein; DPP, dry-cured pork protein; ESP, emulsion-type sausage protein; SP, soy protein; SPP, stewed pork protein.

The values of ACE and Chao in the SP group were significantly lower than in other diet groups. Shannon index of the SP group was also lower than those of the DPP, CPP, and ESP groups (Figure 5A). However, no differences in Simpson index were observed in the cecum. Principal coordinate analysis (PCoA) and multivariate analysis showed that gut microbial communities in mice fed with the processed meat proteins were significantly distinct from those in mice fed with C or SP diet (*P* < 0.05, Figure 5B). Overall, these results reveal that different protein diets could affect gut microbial richness and diversity.

#### Composition of Microbiota

**FIGURE 6:**
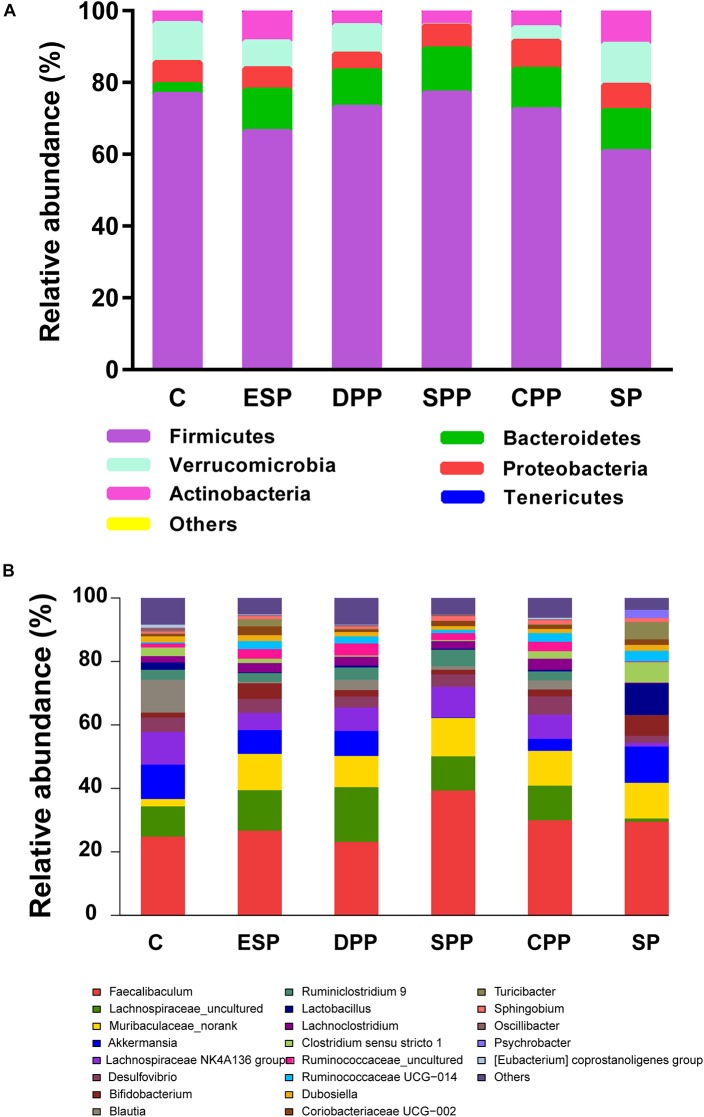
Composition of cecum microbiota. **(A)** At the phyla levels. **(B)** At the genera levels. C, casein; CPP, cooked pork protein; DPP, dry-cured pork protein; ESP, emulsion-type sausage protein; SP, soy protein; SPP, stewed pork protein.

At the phylum level, *Firmicutes* were the most abundant phyla in all diet groups (Figure 6A). Compared with the casein group, the SP, ESP, DPP, SPP, and CPP groups had higher relative abundances of *Bacteroidetes* (2.78, 11.40, 11.65, 10.15, 12.25, and 11.23%, respectively). Lower *Firmicutes* but higher *Actinobacteria* were observed in ESP and SP groups than in other diet groups. The relative abundance of *Verrucomicrobia* was lower in the SPP group than that in the casein, ESP, DPP, CPP, and SP groups (0.20, 10.83, 7.48, 7.91, 3.78, and 11.41%, respectively).

At the genus level, 162 genera were identified in cecal contents (Figure 6B). Compared with the casein group, the processed meat protein and SP groups had lower abundances of *Blautia* but higher abundances of *Muribaculaceae-norank*. The relative abundances of *Lachnospiraceae-uncultured* and *Lachnospiraceae NK4A136 group* were lower in the SP group than in other diet groups. Higher *Faecalibaculum* but lower *Akkermansia* were observed in the SPP group.

**FIGURE 7:**
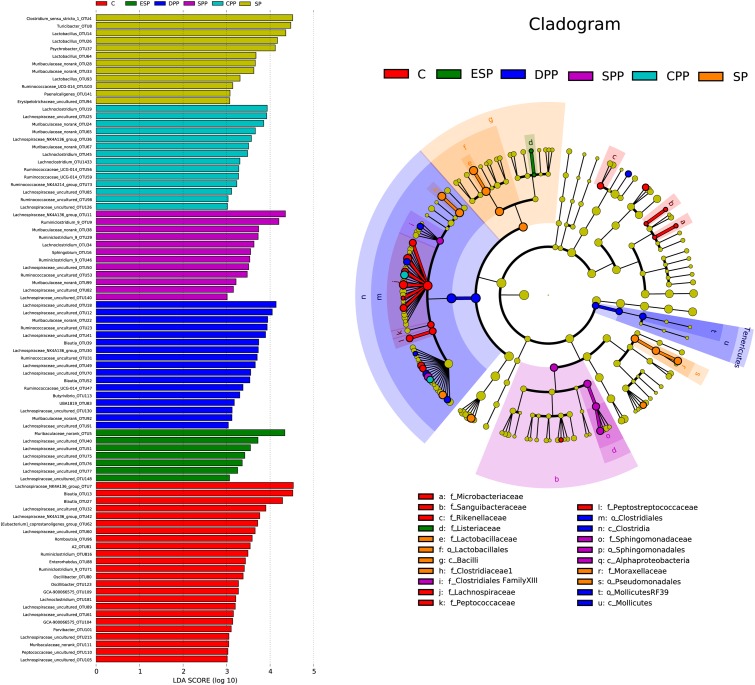
Linear discriminant analysis of caecum microbiota. The left histograms show the LDA scores computed for features at the OTU level. The right biologically cladograms show specific gut microbial taxa from phylum to genus associated with protein diets. C, casein; CPP, cooked pork protein; DPP, dry-cured pork protein; ESP, emulsion-type sausage protein; SP, soy protein; SPP, stewed pork protein.

LEfSe analysis revealed that 86 OTUs, corresponding to 52 genera, significantly differed with diets in the cecum (Figure 7). *Microbacteriaceae*, *Sanguibacteraceae*, *Rikenellaceae*, *Lachnospiraceae*, *Peptococcaceae*, and *Peptostreptococcaceae* were dominant in the casein group. *Lactobacillaceae*, *Clostridiaceae 1*, and *Moraxellaceae* were more abundant in the SP group. *Clostridiales* and *Mollicutes RF39* were more specific for the DPP group, with *Clostridiales Family XIII* and *Sphingomonadaceae* for the SPP group, and *Listeriaceae* for the ESP group. All these results indicated that gut microbiota composition of mice showed a diet-dependent change.

#### Functional Prediction of Microbial Genes

**FIGURE 8:**
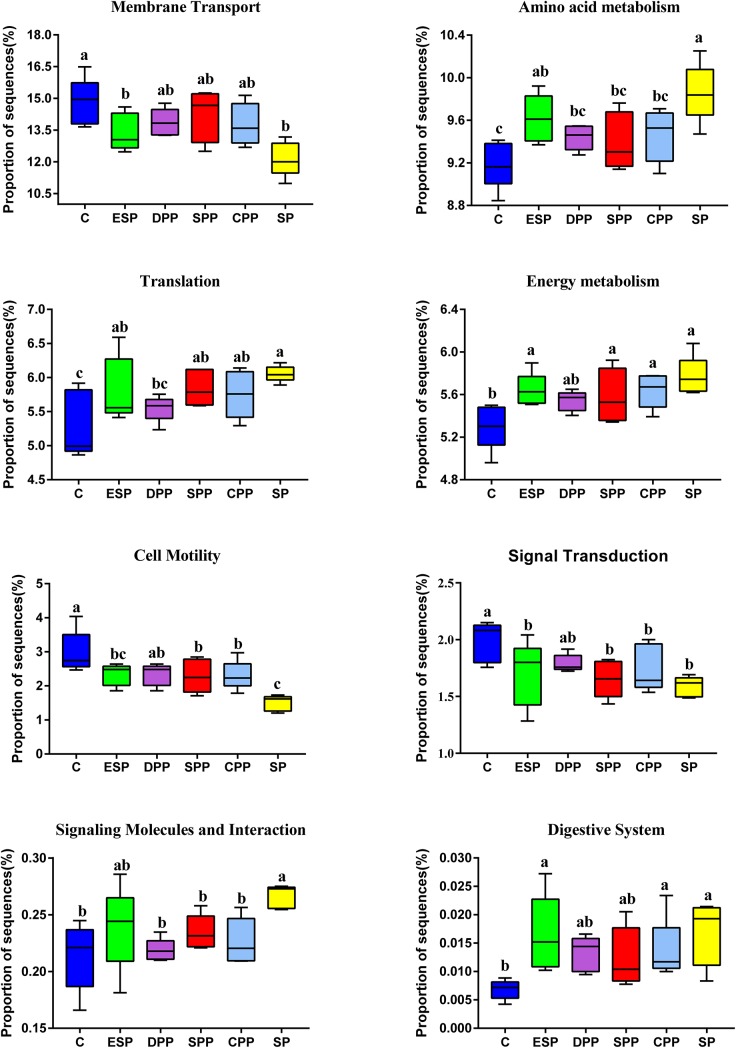
Functional prediction of cecum microbial genes. The data were analyzed by one-way ANOVA, and means were compared by Tukey’s *t*-test. The “a, b, c, d” letters indicate significant differences (*P* < 0.05). C, casein; CPP, cooked pork protein; DPP, dry-cured pork protein; ESP, emulsion-type sausage protein; SP, soy protein; SPP, stewed pork protein.

Compared with the casein diet, the SP and ESP diets upregulated the microbial AA metabolism, energy metabolism, signaling molecules and interaction, translation, and digestive system function but downregulated the microbial membrane transport, signal transduction, and cell motility function (Figure 8).

### Dietary Proteins Affected the Content of SCFAs Through Specific Gut Microbes

SCFAs are one of the important metabolites of gut microbiota. The DPP and ESP groups had lower levels of acetate and total SCFAs than the other groups (*P* < 0.05, Table 2). The processed meat protein groups significantly upregulated the valerate levels compared with non-meat protein groups. The levels of propionate, butyrate, isobutyrate, isovalerate, and branched fatty acids (BCFAs) were much higher in the SPP and SP groups than the other groups (*P* < 0.05).

**TABLE 2:** Short-chain fatty acids levels in cecum contents of mice fed different protein diets (μmol/g).

**Treatment group**	**C**	**ESP**	**DPP**	**SPP**	**CPP**	**SP**
**Cecum contents**						
T-SCFAs	109.8623.95^a^	81.4414.02^b^	92.7924.76^b^	112.4111.29^a^	110.7811.70^a^	111.2116.45^a^
Acetate	99.2220.51^a^	70.2911.37^b^	80.9520.35^b^	96.399.87^a^	97.0810.58^a^	95.7414.38^a^
Propionate	3.401.20^c^	4.071.03^bc^	3.981.32^c^	5.221.44^ab^	4.511.07^bc^	6.241.20^a^
Butyrate	5.372.73^bc^	4.711.29^c^	5.432.71^bc^	7.421.85^a^	6.471.15^bc^	6.731.81^ab^
Valerate	0.530.17^b^	1.040.46^a^	1.110.74^a^	1.170.38^a^	1.180.32^a^	0.630.23^b^
Isobutyrate	0.730.21^bc^	0.720.16^c^	0.690.32^c^	1.120.21^a^	0.810.15^bc^	0.950.35^ab^
Isovalerate	0.610.23^c^	0.610.17^c^	0.630.33^c^	1.080.26^a^	0.740.19^bc^	0.920.31^ab^
BCFAs	1.340.43^c^	1.330.33^c^	1.320.64^c^	2.200.46^a^	1.550.33^bc^	1.870.66^ab^

*T-SCFAs, total short-chain fatty acids; SCFAs, short-chain fatty acids; BCFAs, branched fatty acids. Values are shown as mean ± SD, and means were compared by Tukey’s *t*-test. The “a, b, c” letters indicate significant differences (*P* < 0.05). C, casein; CPP, cooked pork protein; DPP, dry-cured pork protein; ESP, emulsion-type sausage protein; SP, soy protein; SPP, stewed pork protein.*

**FIGURE 9:**
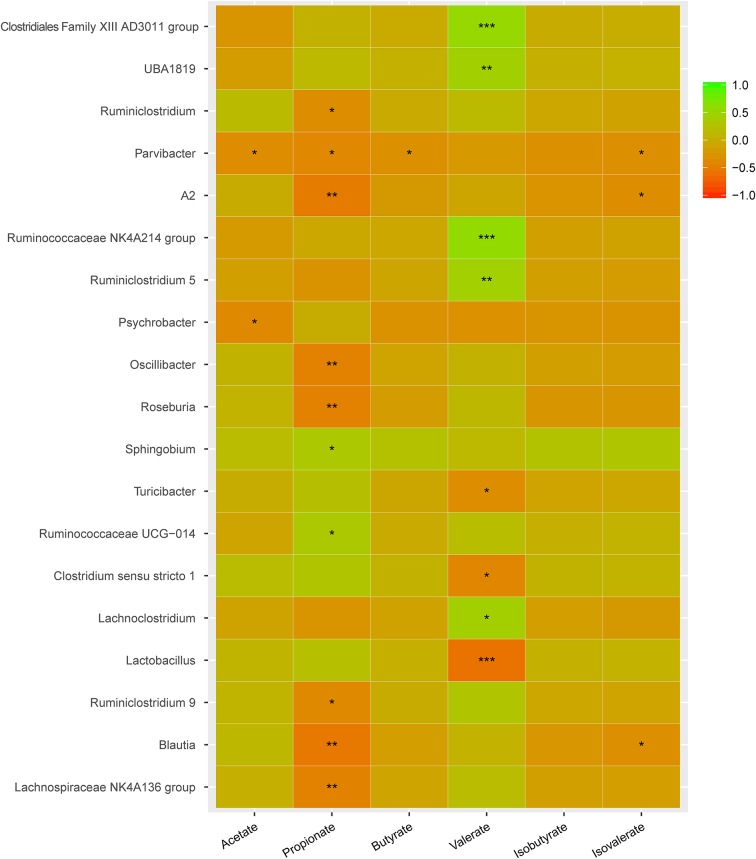
Key genera involved in regulating SCFAs in cecum. Green color represents significant positive correlation and red color represents significant negative correlation, and the independent right color bars depict correlation coefficients between microbiota and SCFAs. Correlation was considered significant when the absolute value of Spearman’s rank correlation coefficient (Spearman’s *r*) was > 0.6 and statistically significant (*P* < 0.05). The significance is indicated: *P < 0.05; **P < 0.01; *** *P* < 0.001.

The associations between changes in the abundance of different genera and concentrations of SCFAs were further revealed by Spearman’s rank correlation analysis (Figure 9). The abundance of *Parvibacter* was negatively correlated with the concentrations of acetate, propionate, butyrate, and isovalerate. The propionate levels were negatively correlated with *Oscillibacter*, *Roseburia*, *Ruminiclostridium 9*, *Blautia*, and *Lachnospiraceae NK4A136 group* but positively with *Sphingobium* and *Ruminococcaceae UCG-014*. The valerate levels were negatively correlated with *Turicibacter*, *Clostridium sensu stricto 1*, and *Lactobacillus* but positively with *Clostridiales Family XIII AD3011 group, BA1819, Ruminococcaceae NK4A214 group, Ruminiclostridium 5*, and *Lachnoclostridium*.

**FIGURE 10:**
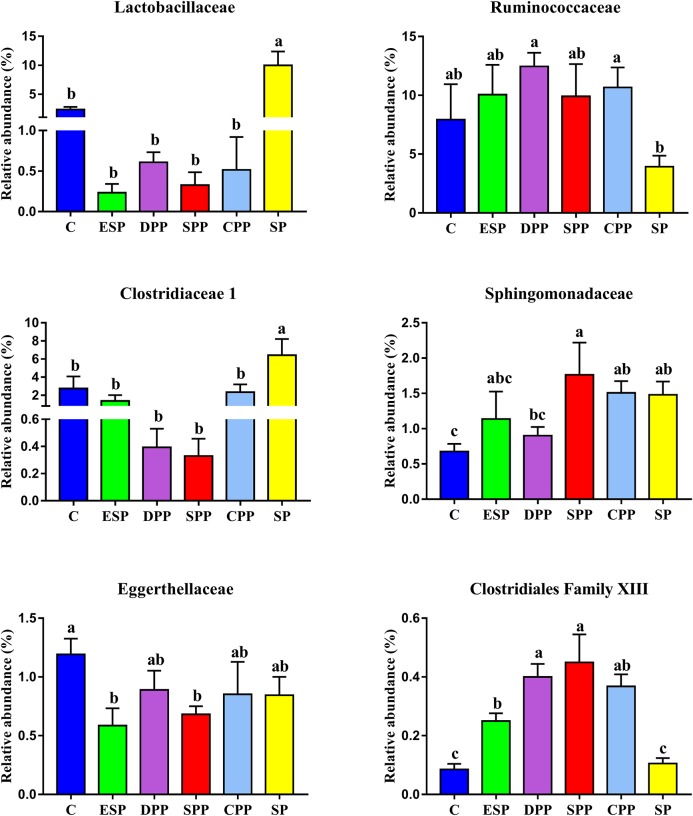
Variations in family significantly associated with SCFAs among dietary protein groups. The data were analyzed by one-way ANOVA, and means were compared by Tukey’s *t*-test. The “a, b, c, d” letters indicate significant differences (*P* < 0.05). C, casein; CPP, cooked pork protein; DPP, dry-cured pork protein; ESP, emulsion-type sausage protein; SP, soy protein; SPP, stewed pork protein.

The genera significantly associated with SCFAs include including *Clostridiaceae 1*, *Eggerthellaceae*, *Erysipelotrichaceae*, *Clostridiales Family XIII*, *Lactobacillaceae*, *Moraxellaceae*, *Ruminococcaceae*, and *Sphingomonadaceae* (Figure 10). *Clostridiaceae 1* and *Lactobacillaceae* were the most abundant in the SP group. However, *Clostridiales Family XIII* was higher in the meat protein groups than in the non-meat protein groups. *Ruminococcaceae* was higher in the DPP and CPP groups than in the SP group. Lower *Eggerthellaceae* was observed in the SPP group than in the casein group, but showed the opposite trend to *Sphingomonadaceae*. These results confirmed that dietary proteins may affect the production of SCFAs by changing specific gut microbes.

## Discussion

AAs are the basic unit of protein, and their most important function is to synthesize proteins. When serum and luminal data are put together, the soy protein seems less bioavailable than casein and processed meat proteins. Proteins from stewed pork seems the least bioavailable among processed meat proteins. This could be related to the differences in protein digestibility, AA transport and metabolism, and gut microbiota activity. Previous studies have shown that the degree of cooking has a significant effect on the protein digestibility and *in vitro* protein digestibility of meat products (Santé-Lhoutellier et al., 2008; Wen et al., 2015). Cooking at lower temperatures induced protein denaturation and reduced disulfide bond formation, which caused the meat proteins to form looser structures and become more susceptible to digestion. However, high-temperature and long-term cooking led to protein aggregation due to higher disulfide bond contents. The gel network formed after chopping, and subsequent heating led to reduced susceptibility to digestion (He J. et al., 2018). The processing methods affect not only the protein digestibility of meat products but also the particle and the peptide fractions released after digestion (Berardo et al., 2017). This can be closely related to protein oxidation and aggregation (Sante-Lhoutellier et al., 2007), which would change the surface hydrophobicity and protein secondary structures (Traore et al., 2011). An *in vitro* study showed that strong protein oxidation occurring during long time cooking reduced the protein digestibility and digestive product profiles (Li et al., 2017). Although protein digestion in the upper digestive tract is relatively efficient, there are a certain amount of proteins, peptides, and AAs entering the large intestine (Bergen and Wu, 2009). They will be fermented by the gut microbiota in the large intestine that can synthesize AAs *de novo*. Their behaviors in the large intestine play an important role in protein metabolism. Therefore, AAs in the large intestine may come from the hydrolysis of undigested dietary proteins and host proteins under the action of microbial protease and peptidase, and from the AAs and peptides that were not absorbed in the small intestine.

In the gut, the microbial growth mainly depends on the remainders of dietary nutrients. The composition of AAs and gut microbiota had similar patterns of change among different dietary protein groups. This indicates that the composition of AAs may determine the composition of gut microbiota to a certain extent. On the contrary, the gut microbiota may also affect the absorption and utilization of host nutrients. The potential biological activities of gut microbiota have a significant impact on host metabolic and nutritional homeostasis, immune system and even brain activity (Zhang and Davies, 2016). The SP and ESP diets upregulated the microbial translation, AA metabolism, digestive system, and energy metabolism function but downregulated the microbial signal transduction and membrane transport function than the casein diet. However, the casein, DPP, SPP, and CPP diets obviously upregulated the microbial cell motility but downregulated the microbial signaling molecules and interaction. SCFAs, as important metabolites, can drive the crosstalk between the host and gut microbiome (Blacher et al., 2017). SCFAs can provide energy for the epithelial cells to stimulate cell proliferation, differentiation, and maturation, and reduce cell apoptosis (Koh et al., 2016). They can also bond to G protein coupled receptors and initiate host immunity and gut motility. Some studies have pointed out that acetate, propionate, and butyrate have anti-inflammatory effects (Arpaia et al., 2013). The SPP groups may increase contents of acetate, propionate, butyrate, and isovalerate by decreasing *Eggerthellaceae* abundance, and increase propionate level by increasing the *Sphingomonadaceae* or decreasing *Lactobacillaceae*. However, the SPP decreased the relative abundance of *Akkermansia* than the other diet groups. *Akkermansia* is an important genus of the *Verrucomicrobia* phylum. It is abundant in the small intestinal epithelial crypts and mucus layer (Reunanen et al., 2015). Several studies showed that *Akkermansia muciniphila* abundance was related to insulin sensitivity, and metabolic status (Anonye, 2017). The abundance of *A. muciniphila* was observed lower in the intestinal tract of IBD patients. Other studies showed that *A. muciniphila* can decrease the high-fat-diet induced burden of the mice (Zhao et al., 2016). Therefore, the effects of SPP diet on the health remain to be further investigated.

AA composition of dietary proteins has a great impact on the development of animals. Tryptophan is a biosynthetic precursor of numerous microbial and host metabolites (Alkhalaf and Ryan, 2015). Arginine and ornithine play a critical role in the division, differentiation, and repair of intestinal epithelial cells. They can also enhance the function of the intestinal barrier (Wang et al., 2009). Glutamine has a function in regulating growth performance and maintaining gut health (Wu et al., 2011). In the present study, the SP group significantly decreased the crypt depth but increased the villi height and the V/C ratio in duodenum compared with the other groups. The small intestines of mice fed with the SP diet were longer. This indicates that more AAs might have been utilized for the growth and development of tissues, which may partly explain why the SP group had lower content of AAs in gut and serum. The villi height, crypt depth, and V/C ratio are important indices to evaluate the digestibility of the small intestine. The normal structure and function of the villus and crypt are the physiological basis to ensure the digestion and absorption of nutrients and the normal growth of the body (Vesna et al., 2011). The increase in villi height may be attributed to an increasing number of mature cells in intestinal villi with enlarged area of wrinkled wall and enhanced absorption capacity of epithelial cells. The decrease in crypt depth should be ascribed to increasing maturation rate and secretion capacity of epithelial cells. The ratios reflect the net absorption capacity of the small intestine, with the higher the ratio, the stronger the absorption and transport capacity of intestinal epithelial cells. The cecum of germ-free rats was enlarged up to 10 times of its normal size and gastric emptying and intestinal transit were delayed than conventionally raised animals (Barbara et al., 2005). In the present study, the microbial abundance of the SP group in cecum was the lowest but the cecum weight was the highest. Therefore, soy protein diet was beneficial for tissue development and morphology but may reduce intestinal peristalsis. There are also many other factors that can affect the peristalsis, such as enteric nervous system and intestinal hormones (Dey et al., 2015). Further studies are needed because this relationship is very complicated.

In summary, different protein diets significantly affected AA profiles in gut contents and serum, and the processed meat proteins and casein showed higher bioavailability than SP. Proteins from stewed pork seems the least bioavailable among processed meat proteins. The composition of AAs in processed meat protein diets was different from the casein or SP diet, which determined the composition and function of gut microbiota to a certain extent. SPP diet increased the production of SCFAs by specific gut microbiota, but it also decreased the abundance of beneficial bacteria than the other diets. Soy protein diet was beneficial for tissue development and microbial metabolism but may reduce intestinal peristalsis compared with casein and processed meat protein diets. This study provides useful information on interactions among protein diets, gut microbiota, and host.

## Data Availability Statement

The datasets generated for this study can be found in the NCBI Sequence Read Archive under accession code SRP250221.

## Ethics Statement

The animal study was reviewed and approved by Ethical Committee of Experimental Animal Center of the Nanjing Agricultural University.

## Author Contributions

CL and YX designed the research, acquired all the raw data, wrote the manuscript, and performed the data analysis. YX and CW completed the experimental animal feeding. CL, DZ, and GZ provided critical comments on the manuscript. All authors reviewed the manuscript.

## Conflict of Interest

The authors declare that the research was conducted in the absence of any commercial or financial relationships that could be construed as a potential conflict of interest.
