# Supplementary material for: Processing Method Altered Mouse Intestinal Morphology and Microbial Composition by Affecting Digestion of Meat Proteins
Source: Front Microbiol. 2020 Apr 8;11:511. doi: 10.3389/fmicb.2020.00511 (PMC7156556; doi:10.3389/fmicb.2020.00511)
Supplement: Supplementary file 1 [file Table_1.DOCX]

**Table S1. Amino acid composition in serum (mmol/L).**

|  | ESP | SP | DPP | SPP | CPP | C |
| --- | --- | --- | --- | --- | --- | --- |
| Asp | 0.017±0.004^ab^ | 0.015±0.004^b^ | 0.017±0.003^ab^ | 0.016±0.003^b^ | 0.022±0.006^a^ | 0.016±0.003^b^ |
| Glu | 0.060±0.011^b^ | 0.051±0.011^b^ | 0.073±0.008^a^ | 0.053±0.009^b^ | 0.074±0.012^a^ | 0.063±0.013^ab^ |
| Asn | 0.047±0.011 | 0.043±0.010 | 0.051±0.012 | 0.042±0.007 | 0.051±0.007 | 0.060±0.028 |
| Ser | 0.150±0.026^ab^ | 0.102±0.017^c^ | 0.177±0.042^a^ | 0.133±0.031b^c^ | 0.179±0.016^a^ | 0.176±0.047^a^ |
| His | 0.050±0.007^c^ | 0.050±0.004^c^ | 0.042±0.009^c^ | 0.053±0.008^c^ | 0.118±0.026^a^ | 0.098±0.017^b^ |
| Gln | 0.384±0.079^b^ | 0.393±0.046^b^ | 0.474±0.069^a^ | 0.396±0.063^b^ | 0.446±0.033^ab^ | 0.475±0.071^a^ |
| Arg | 0.211±0.050^b^ | 0.143±0.034^c^ | 0.245±0.066^b^ | 0.223±0.028^b^ | 0.258±0.042^a^ | 0.311±0.058^a^ |
| Gly | 0.467±0.081^a^ | 0.214±0.031^b^ | 0.605±0.264^a^ | 0.302±0.052^b^ | 0.477±0.087^a^ | 0.242±0.058^b^ |
| Thr | 0.291±0.067^b^ | 0.150±0.031^c^ | 0.260±0.072^b^ | 0.277±0.039^b^ | 0.321±0.060^b^ | 0.401±0.106^a^ |
| Tyr | 0.119±0.025^bc^ | 0.101±0.022^c^ | 0.129±0.026^bc^ | 0.119±0.010^bc^ | 0.146±0.023^b^ | 0.221±0.071^a^ |
| Ala | 0.386±0.098^bc^ | 0.296±0.046^c^ | 0.498±0.129^a^ | 0.368±0.068^bc^ | 0.465±0.074^ab^ | 0.342±0.116^c^ |
| Trp | 0.087±0.017^ab^ | 0.070±0.013^c^ | 0.098±0.014^a^ | 0.073±0.008^bc^ | 0.085±0.016^abc^ | 0.081±0.015^bc^ |
| Met | 0.096±0.061^a^ | 0.327±0.045^b^ | 0.412±0.111^a^ | 0.312±0.030^b^ | 0.408±0.058^a^ | 0.271±0.050^b^ |
| Val | 0.195±0.053^bc^ | 0.122±0.027^c^ | 0.310±0.128^a^ | 0.170±0.030^bc^ | 0.233±0.049^ab^ | 0.229±0.074^b^ |
| Phe | 0.058±0.021^bc^ | 0.032±0.008^c^ | 0.087±0.031^a^ | 0.042±0.020^c^ | 0.083±0.020^ab^ | 0.074±0.040^ab^ |
| Ile | 0.085±0.027^abc^ | 0.061±0.015^c^ | 0.110±0.034^a^ | 0.082±0.014^bc^ | 0.103±0.021^ab^ | 0.095±0.032^ab^ |
| Leu | 0.156±0.040^abc^ | 0.115±0.023^c^ | 0.185±0.053^a^ | 0.135±0.018^ab^ | 0.168±0.030^ab^ | 0.143±0.042^bc^ |
| Lys | 0.267±0.070^b^ | 0.160±0.055^c^ | 0.358±0.127^ab^ | 0.315±0.044^b^ | 0.353±0.084^ab^ | 0.426±0.086^a^ |
| Total AAs | 3.127±0.560^b^ | 2.445±0.325^c^ | 4.131±1.024^a^ | 3.112±0.337^b^ | 3.991±0.485^a^ | 3.725±0.749^a^ |

Values are shown as mean ± SD. The data were analyzed by one-way ANOVA, and means were compared by Tukey’s t test. The “a, b, c” letters indicate significant differences (*P*< 0.05). C, casein; CPP, cooked pork protein; DPP, dry-cured pork protein; ESP, emulsion-type sausage protein; SP, soy protein; SPP, stewed pork protein.
