# Supplementary material for: Processing Method Altered Mouse Intestinal Morphology and Microbial Composition by Affecting Digestion of Meat Proteins
Source: Front Microbiol. 2020 Apr 8;11:511. doi: 10.3389/fmicb.2020.00511 (PMC7156556; doi:10.3389/fmicb.2020.00511)
Supplement: Supplementary file 2 [file Table_2.DOCX]

**Table S2. Amino acid composition in duodenal contents (g/kg).**

|  | ESP | SP | DPP | SPP | CPP | C |
| --- | --- | --- | --- | --- | --- | --- |
| Asp | 0.578±0.240^ab^ | 0.373±0.114^b^ | 0.652±0.098^a^ | 0.779±0.251^a^ | 0.750±0.290^a^ | 0.356±0.214^b^ |
| Glu | 0.669±0.100^b^c | 0.854±0.165^a^ | 0.838±0.111^a^ | 0.678±0.150^bc^ | 0.556±0.105^c^ | 0.821±0.148^ab^ |
| Asn | 0.005±0.003^c^ | 0.012±0.008^bc^ | 0.013±0.006^b^ | 0.013±0.006^b^ | 0.004±0.003^c^ | 0.026±0.009^a^ |
| Ser | 0.044±0.013^c^ | 0.047±0.016^c^ | 0.065±0.019^ab^ | 0.055±0.016^bc^ | 0.042±0.009^c^ | 0.076±0.017^a^ |
| His | 0.046±0.061^b^ | 0.039±0.013^b^ | 0.016±0.014^b^ | 0.285±0.182^a^ | 0.019±0.018^b^ | 0.078±0.052^b^ |
| Gln | 0.012±0.009^b^ | 0.020±0.006^b^ | 0.021±0.008^b^ | 0.016±0.017^b^ | 0.012±0.005^b^ | 0.041±0.016^a^ |
| Arg | 0.197±0.048^bcd^ | 0.182±0.061^cd^ | 0.269±0.070^b^ | 0.362±0.111^a^ | 0.168±0.043^d^ | 0.261±0.078^bc^ |
| Gly | 0.054±0.020^c^ | 0.136±0.044^a^ | 0.095±0.036^b^ | 0.104±0.038^ab^ | 0.052±0.016^c^ | 0.077±0.021^bc^ |
| Thr | 0.046±0.017^cd^ | 0.071±0.023^bc^ | 0.103±0.034^a^ | 0.078±0.018^ab^ | 0.041±0.010^d^ | 0.078±0.029^ab^ |
| Tyr | 0.119±0.032^b^ | 0.131±0.046^b^ | 0.186±0.062^a^ | 0.191±0.067^a^ | 0.092±0.013^b^ | 0.123±0.041^b^ |
| Ala | 0.167±0.045^bc^ | 0.260±0.109^ab^ | 0.333±0.136^a^ | 0.301±0.081^a^ | 0.141±0.038^c^ | 0.242±0.107^abc^ |
| Trp | 0.046±0.019^b^ | 0.060±0.025^b^ | 0.065±0.016^b^ | 0.112±0.019^a^ | 0.057±0.027^b^ | 0.049±0.012^b^ |
| Met | 0.057±0.019^bc^ | 0.058±0.029^bc^ | 0.107±0.044^a^ | 0.107±0.035^a^ | 0.036±0.015^c^ | 0.075±0.044^ab^ |
| Val | 0.087±0.044^cd^ | 0.120±0.056^bcd^ | 0.179±0.072^ab^ | 0.180±0.050^a^ | 0.069±0.033^d^ | 0.131±0.058^abc^ |
| Phe | 0.108±0.033^b^ | 0.088±0.033^b^ | 0.210±0.068^a^ | 0.210±0.061^a^ | 0.123±0.033^b^ | 0.098±0.049^b^ |
| Ile | 0.065±0.029^bc^ | 0.086±0.047^bc^ | 0.147±0.072^a^ | 0.146±0.050^a^ | 0.043±0.014^c^ | 0.107±0.042^ab^ |
| Leu | 0.098±0.038^c^ | 0.120±0.056^bc^ | 0.208±0.110^a^ | 0.215±0.067^a^ | 0.078±0.026^c^ | 0.183±0.067^ab^ |
| Lys | 0.321±0.108^b^ | 0.312±0.113^b^ | 0.559±0.208^a^ | 0.586±0.123^a^ | 0.268±0.092^b^ | 0.388±0.166^b^ |
| Total AAs | 2.718±0.492^b^ | 2.967±0.775^b^ | 4.067±1.029^a^ | 4.419±0.764^a^ | 2.551±0.525^b^ | 3.210±0.873^b^ |

Values are shown as mean ± SD. The data were analyzed by one-way ANOVA, and means were compared by Tukey’s t test. The “a, b, c” letters indicate significant differences (*P*< 0.05). C, casein; CPP, cooked pork protein; DPP, dry-cured pork protein; ESP, emulsion-type sausage protein; SP, soy protein; SPP, stewed pork protein.
