# Supplementary material for: Processing Method Altered Mouse Intestinal Morphology and Microbial Composition by Affecting Digestion of Meat Proteins
Source: Front Microbiol. 2020 Apr 8;11:511. doi: 10.3389/fmicb.2020.00511 (PMC7156556; doi:10.3389/fmicb.2020.00511)
Supplement: Supplementary file 3 [file Table_3.DOCX]

**Table S3. Amino acid composition in jejunal contents (g/kg).**

|  | ESP | SP | DPP | SPP | CPP | C |
| --- | --- | --- | --- | --- | --- | --- |
| Asp | 0.364±0.093^bc^ | 0.372±0.079^ab^c | 0.440±0.110^ab^ | 0.463±0.113^ab^ | 0.471±0.092^a^ | 0.273±0.038^c^ |
| Glu | 0.565±0.110^b^ | 0.703±0.190^ab^ | 0.764±0.231^ab^ | 0.790±0.158^a^ | 0.889±0.230^a^ | 0.563±0.175^b^ |
| Asn | 0.083±0.048^b^ | 0.204±0.060^a^ | 0.170±0.091^a^ | 0.143±0.034^ab^ | 0.178±0.065^a^ | 0.160±0.056^a^ |
| Ser | 0.139±0.060^c^ | 0.240±0.089^ab^ | 0.241±0.112^ab^ | 0.273±0.108^a^ | 0.205±0.066^abc^ | 0.173±0.059^bc^ |
| His | 0.550±0.303^b^ | 0.733±0.233^ab^ | 0.813±0.281^ab^ | 0.946±0.346^a^ | 0.679±0.235^ab^ | 0.606±0.209^b^ |
| Gln | 0.013±0.026^ab^ | 0.032±0.028^ab^ | 0.041±0.034^a^ | 0.024±0.041^ab^ | 0.016±0.022^ab^ | 0.001±0.001^b^ |
| Arg | 0.195±0.047^bc^ | 0.177±0.061^c^ | 0.266±0.067^ab^ | 0.343±0.085^a^ | 0.169±0.045^c^ | 0.250±0.101^bc^ |
| Gly | 0.054±0.020^c^ | 0.134±0.049^a^ | 0.095±0.036^b^ | 0.096±0.023^b^ | 0.052±0.015c | 0.067±0.031^bc^ |
| Thr | 0.046±0.016^cd^ | 0.069±0.024^bc^ | 0.102±0.033^a^ | 0.079±0.016^ab^ | 0.041±0.009^d^ | 0.072±0.031^bc^ |
| Tyr | 0.119±0.032^c^ | 0.131±0.045^bc^ | 0.185±0.062^a^ | 0.193±0.064^ab^ | 0.088±0.017^c^ | 0.116±0.048^c^ |
| Ala | 0.179±0.059^bc^ | 0.259±0.111^ab^ | 0.332±0.139^a^ | 0.304±0.073^a^ | 0.141±0.034^c^ | 0.229±0.113^abc^ |
| Trp | 0.046±0.018^bc^ | 0.060±0.026^bc^ | 0.069±0.022^b^ | 0.110±0.010^a^ | 0.058±0.026^bc^ | 0.043±0.018^c^ |
| Met | 0.056±0.019^b^ | 0.059±0.029^b^ | 0.106±0.044^a^ | 0.108±0.03^4a^ | 0.036±0.015^b^ | 0.070±0.047^ab^ |
| Val | 0.086±0.043^c^ | 0.120±0.055^bc^ | 0.178±0.071^ab^ | 0.183±0.04^8a^ | 0.069±0.033^c^ | 0.125±0.066^abc^ |
| Phe | 0.275±0.106^b^ | 0.439±0.174^a^ | 0.414±0.116^ab^ | 0.399±0.124^ab^ | 0.398±0.194^ab^ | 0.325±0.105^ab^ |
| Ile | 0.188±0.068^b^ | 0.333±0.105^a^ | 0.309±0.102^a^ | 0.304±0.115^a^ | 0.266±0.111^ab^ | 0.261±0.09^0ab^ |
| Leu | 0.426±0.190^b^ | 0.715±0.275^a^ | 0.654±0.202^ab^ | 0.645±0.245^ab^ | 0.541±0.244^ab^ | 0.508±0.185^ab^ |
| Lys | 0.318±0.106^b^ | 0.306±0.113^b^ | 0.555±0.204^a^ | 0.556±0.156^a^ | 0.268±0.087^b^ | 0.363±0.175^b^ |
| Total AAs | 3.703±0.990^c^ | 5.060±1.265^ab^ | 5.736±1.322^b^ | 5.946±1.229^a^ | 4.567±0.826^bc^ | 4.204±1.030^bc^ |

Values are shown as mean ± SD. The data were analyzed by one-way ANOVA, and means were compared by Tukey’s t test. The “a, b, c” letters indicate significant differences (*P*< 0.05). C, casein; CPP, cooked pork protein; DPP, dry-cured pork protein; ESP, emulsion-type sausage protein; SP, soy protein; SPP, stewed pork protein.
