# Supplementary material for: Processing Method Altered Mouse Intestinal Morphology and Microbial Composition by Affecting Digestion of Meat Proteins
Source: Front Microbiol. 2020 Apr 8;11:511. doi: 10.3389/fmicb.2020.00511 (PMC7156556; doi:10.3389/fmicb.2020.00511)
Supplement: Supplementary file 4 [file Table_4.DOCX]

**Table S4. Amino acid composition in cecal contents (g/kg).**

|  | ESP | SP | DPP | SPP | CPP | C |
| --- | --- | --- | --- | --- | --- | --- |
| Asp | 0.690±0.359^a^ | 0.377±0.112^b^ | 0.658±0.076^a^ | 0.795±0.268^a^ | 0.742±0.275^a^ | 0.363±0.211^b^ |
| Glu | 0.733±0.160^a^ | 0.851±0.155^a^ | 0.850±0.121^a^ | 0.686±0.132^ab^ | 0.556±0.090^b^ | 0.799±0.204^a^ |
| Asn | 0.005±0.003^cd^ | 0.012±0.005^bc^ | 0.013±0.006^b^ | 0.013±0.008^b^ | 0.004±0.003^d^ | 0.027±0.009^a^ |
| Ser | 0.046±0.014^b^ | 0.047±0.014^b^ | 0.066±0.020^a^ | 0.060±0.020^ab^ | 0.042±0.009^b^ | 0.078±0.016^a^ |
| His | 0.013±0.012^b^ | 0.037±0.013^b^ | 0.016±0.013^b^ | 0.038±0.040^b^ | 0.018±0.018^b^ | 0.079±0.052^a^ |
| Gln | 0.015±0.008^b^ | 0.019±0.006^b^ | 0.022±0.008^b^ | 0.019±0.010^b^ | 0.012±0.006^b^ | 0.043±0.016^a^ |
| Arg | 0.205±0.049^bc^ | 0.184±0.060^c^ | 0.297±0.100^a^ | 0.341±0.087^a^ | 0.168±0.049^c^ | 0.270±0.082^ab^ |
| Gly | 0.057±0.023^c^ | 0.138±0.046^a^ | 0.096±0.034^b^ | 0.105±0.034^ab^ | 0.051±0.015^c^ | 0.079±0.020^bc^ |
| Thr | 0.049±0.018^cd^ | 0.072±0.023^bc^ | 0.105±0.034^a^ | 0.085±0.024^ab^ | 0.040±0.009^d^ | 0.080±0.028^ab^ |
| Tyr | 0.125±0.036^c^ | 0.133±0.045^bc^ | 0.190±0.067^a^ | 0.177±0.049^ab^ | 0.092±0.014^c^ | 0.127±0.042^bc^ |
| Ala | 0.189±0.063^bc^ | 0.266±0.120^ab^ | 0.339±0.141^a^ | 0.305±0.078^a^ | 0.139±0.032^c^ | 0.248±0.105^ab^ |
| Trp | 0.048±0.019^b^ | 0.066±0.018^b^ | 0.066±0.017^b^ | 0.114±0.015^a^ | 0.051±0.019^b^ | 0.050±0.011^b^ |
| Met | 0.059±0.019^bc^ | 0.059±0.028^bc^ | 0.109±0.046^a^ | 0.109±0.037^a^ | 0.036±0.015^c^ | 0.091±0.025^ab^ |
| Val | 0.090±0.045^cd^ | 0.122±0.058^bc^ | 0.182±0.074^ab^ | 0.184±0.052^a^ | 0.061±0.025^d^ | 0.141±0.045^abc^ |
| Phe | 0.124±0.048^b^ | 0.089±0.033^b^ | 0.213±0.068^a^ | 0.213±0.062^a^ | 0.121±0.030^b^ | 0.100±0.049^b^ |
| Ile | 0.068±0.029^bc^ | 0.088±0.048^bc^ | 0.150±0.073^a^ | 0.149±0.053^a^ | 0.043±0.013^c^ | 0.110±0.043^ab^ |
| Leu | 0.102±0.038^c^ | 0.121±0.056^bc^ | 0.212±0.110^a^ | 0.219±0.070^a^ | 0.078±0.026^c^ | 0.188±0.067^ab^ |
| Lys | 0.334±0.110^b^ | 0.316±0.112^b^ | 0.571±0.226^a^ | 0.561±0.176^a^ | 0.245±0.066^b^ | 0.397±0.166^ab^ |
| Total AAs | 2.951±0.711^b^ | 2.998±0.743^b^ | 4.153±1.111^a^ | 4.171±0.817^a^ | 2.501±0.465^b^ | 3.270±0.946^b^ |

Values are shown as mean ± SD. The data were analyzed by one-way ANOVA, and means were compared by Tukey’s t test. The “a, b, c” letters indicate significant differences (*P*< 0.05). C, casein; CPP, cooked pork protein; DPP, dry-cured pork protein; ESP, emulsion-type sausage protein; SP, soy protein; SPP, stewed pork protein.
