# Supplementary material for: Processing Method Altered Mouse Intestinal Morphology and Microbial Composition by Affecting Digestion of Meat Proteins
Source: Front Microbiol. 2020 Apr 8;11:511. doi: 10.3389/fmicb.2020.00511 (PMC7156556; doi:10.3389/fmicb.2020.00511)
Supplement: Supplementary file 5 [file Table_5.DOCX]

**Table S5. Amino acid composition in colonic contents (g/kg).**

|  | ESP | SP | DPP | SPP | CPP | C |
| --- | --- | --- | --- | --- | --- | --- |
| Asp | 0.880±0.538^a^ | 0.247±0.145^c^ | 0.691±0.330^ab^ | 0.799±0.419^a^ | 0.788±0.190^a^ | 0.333±0.203^bc^ |
| Glu | 0.348±0.076^a^ | 0.216±0.062^b^ | 0.352±0.054^a^ | 0.336±0.063^a^ | 0.326±0.109^a^ | 0.413±0.136^a^ |
| Asn | 0.011±0.011^ab^ | 0.003±0.001^c^ | 0.004±0.001^bc^ | 0.008±0.006^abc^ | 0.004±0.001^bc^ | 0.012±0.009^a^ |
| Ser | 0.060±0.015^b^ | 0.029±0.013^c^ | 0.044±0.011^bc^ | 0.056±0.017^b^ | 0.049±0.013^bc^ | 0.094±0.041^a^ |
| His | 0.039±0.018^ab^ | 0.046±0.016^ab^ | 0.042±0.029^ab^ | 0.072±0.044^a^ | 0.029±0.017^b^ | 0.074±0.045^a^ |
| Gln | 0.024±0.013^ab^ | 0.015±0.003^b^ | 0.022±0.011^ab^ | 0.036±0.021^a^ | 0.022±0.009^ab^ | 0.029±0.016^ab^ |
| Arg | 0.110±0.035^ab^ | 0.038±0.009^c^ | 0.100±0.028^ab^ | 0.151±0.081^a^ | 0.084±0.014^bc^ | 0.124±0.053^ab^ |
| Gly | 0.047±0.015 | 0.056±0.020 | 0.052±0.012 | 0.060±0.022 | 0.063±0.026 | 0.061±0.026 |
| Thr | 0.050±0.012^bc^ | 0.030±0.009^c^ | 0.053±0.014^b^ | 0.049±0.010^bc^ | 0.048±0.017^bc^ | 0.087±0.037^a^ |
| Tyr | 0.099±0.032^b^ | 0.042±0.014^b^ | 0.066±0.015^b^ | 0.104±0.055^b^ | 0.071±0.016^b^ | 0.136±0.046^a^ |
| Ala | 0.139±0.049^ab^ | 0.087±0.018^b^ | 0.150±0.049^a^ | 0.163±0.066^a^ | 0.140±0.046^ab^ | 0.178±0.053^a^ |
| Trp | 0.034±0.030^b^ | 0.020±0.019^b^ | 0.033±0.017^b^ | 0.070±0.042^a^ | 0.074±0.027^a^ | 0.028±0.022^b^ |
| Met | 0.047±0.021^ab^ | 0.021±0.005^c^ | 0.039±0.012^abc^ | 0.057±0.033^a^ | 0.027±0.015^bc^ | 0.059±0.030^a^ |
| Val | 0.113±0.032^ab^ | 0.044±0.013^c^ | 0.058±0.026^bc^ | 0.115±0.084^ab^ | 0.044±0.020^c^ | 0.137±0.080^a^ |
| Phe | 0.078±0.030^abc^ | 0.044±0.016^c^ | 0.098±0.025^ab^ | 0.110±0.039^a^ | 0.064±0.027^bc^ | 0.088±0.046^ab^ |
| Ile | 0.057±0.026^bc^ | 0.019±0.008^d^ | 0.037±0.013^cd^ | 0.079±0.049^ab^ | 0.040±0.014^cd^ | 0.093±0.044^a^ |
| Leu | 0.075±0.037^bc^ | 0.029±0.014^c^ | 0.071±0.033^c^ | 0.137±0.092^ab^ | 0.061±0.023^c^ | 0.144±0.079^a^ |
| Lys | 0.194±0.070^bc^ | 0.111±0.027^c^ | 0.237±0.077^ab^ | 0.317±0.185^a^ | 0.164±0.045^bc^ | 0.248±0.087^ab^ |
| Total AAs | 2.405±0.729^a^ | 1.097±0.203^b^ | 2.152±0.404^a^ | 2.718±1.087^a^ | 2.098±0.426^a^ | 2.339±0.832^a^ |

Values are shown as mean ± SD. The data were analyzed by one-way ANOVA, and means were compared by Tukey’s t test. The “a, b, c” letters indicate significant differences (*P*< 0.05). C, casein; CPP, cooked pork protein; DPP, dry-cured pork protein; ESP, emulsion-type sausage protein; SP, soy protein; SPP, stewed pork protein.
